# Supplementary material for: Deletion of wfs1 Impairs Oligodendrocyte Precursor Cells Dorsal Distribution and Myelination Through the wfs1-hmgcs1 Axis in Zebrafish
Source: Biology (Basel). 2026 Aug 16;15(16):1402. doi: 10.3390/biology15161402 (PMC13510151; doi:10.3390/biology15161402)
Supplement: Supplementary file 1 [file biology-15-01402-s001.zip › File S1-Transcriptome sequencing data and differentially expressed genes (DEGs) in wfs1-- zebrafish at 5 dpf/1.GO/wfs1KOvsWT/GSEA/WT/CELL_CORTEX_PART/CELL_CORTEX_PART(GO_0044448).html]

Details for gene set CELL\_CORTEX\_PART(GO:0044448)[GSEA]


|  || Dataset | deg.sample |
| Phenotype | sample.cls |
| Upregulated in class | WT |
| GeneSet | CELL\_CORTEX\_PART(GO:0044448) |
| Enrichment Score (ES) | -0.4128824 |
| Normalized Enrichment Score (NES) | -1.8317564 |
| Nominal p-value | 0.0 |
| FDR q-value | 0.22709328 |
| FWER p-Value | 0.588 |
Table: GSEA Results Summary

  

Fig 1: Enrichment plot: CELL\_CORTEX\_PART(GO:0044448)
  
Profile of the Running ES Score & Positions of GeneSet Members on the Rank Ordered List

  

| PROBE | DESCRIPTION   (from dataset) | GENE SYMBOL | GENE\_TITLE | RANK IN GENE LIST | RANK METRIC SCORE | RUNNING ES | CORE ENRICHMENT || 1 | 641575 | actn2b |  |  | 1758 | 1.499 | -0.0209 | No |
| 2 | 572002 | erc2 |  |  | 3148 | 1.002 | -0.0416 | No |
| 3 | 100002992 | apnl |  |  | 5004 | 0.680 | -0.0843 | No |
| 4 | 557015 | mlphb |  |  | 5669 | 0.620 | -0.0910 | No |
| 5 | 563414 | rims3 |  |  | 5917 | 0.595 | -0.0851 | No |
| 6 | 556486 | sept4a |  |  | 6511 | 0.536 | -0.0915 | No |
| 7 | 560668 | phldb2b |  |  | 6634 | 0.525 | -0.0833 | No |
| 8 | 335987 | gypc |  |  | 6703 | 0.519 | -0.0735 | No |
| 9 | 568029 | exoc8 |  |  | 7083 | 0.485 | -0.0743 | No |
| 10 | 553729 | dbnlb |  |  | 8709 | 0.387 | -0.1165 | No |
| 11 | 394136 | sept7a |  |  | 9042 | 0.365 | -0.1186 | No |
| 12 | 570807 | phldb2a |  |  | 9558 | 0.330 | -0.1273 | No |
| 13 | 571702 | sept8b |  |  | 9890 | 0.310 | -0.1306 | No |
| 14 | 795670 | llgl2 |  |  | 9936 | 0.307 | -0.1249 | No |
| 15 | 569366 | tnfaip2b |  |  | 10038 | 0.301 | -0.1212 | No |
| 16 | 560400 | actn1 |  |  | 10299 | 0.285 | -0.1229 | No |
| 17 | 568203 | arv1 |  |  | 10861 | 0.251 | -0.1347 | No |
| 18 | 393672 | rims2a |  |  | 11127 | 0.238 | -0.1376 | No |
| 19 | 322338 | exoc1 |  |  | 11688 | 0.207 | -0.1505 | No |
| 20 | 559035 | shroom4 |  |  | 11768 | 0.202 | -0.1483 | No |
| 21 | 447899 | rtkn2b |  |  | 11830 | 0.199 | -0.1457 | No |
| 22 | 562517 | anln |  |  | 12145 | 0.182 | -0.1514 | No |
| 23 | 561153 | dbn1 |  |  | 12276 | 0.175 | -0.1515 | No |
| 24 | 321155 | si:dkey-238i5.2 |  |  | 12314 | 0.173 | -0.1487 | No |
| 25 | 445325 | sept5b |  |  | 12645 | 0.157 | -0.1554 | No |
| 26 | 559010 | exoc5 |  |  | 13041 | 0.138 | -0.1647 | No |
| 27 | 559364 | shroom1 |  |  | 13454 | 0.118 | -0.1749 | No |
| 28 | 101180909 | shroom3 |  |  | 14104 | 0.087 | -0.1934 | No |
| 29 | 559866 | exoc4 |  |  | 14147 | 0.085 | -0.1927 | No |
| 30 | 373082 | gys2 |  |  | 14567 | 0.066 | -0.2044 | No |
| 31 | 562077 | clasp1a |  |  | 14726 | 0.059 | -0.2080 | No |
| 32 | 323457 | sept2 |  |  | 14860 | 0.053 | -0.2110 | No |
| 33 | 100149357 | rims1a |  |  | 15345 | 0.030 | -0.2255 | No |
| 34 | 335925 | sept5a |  |  | 15509 | 0.022 | -0.2301 | No |
| 35 | 568486 | LOC568486 |  |  | 15988 | -0.001 | -0.2451 | No |
| 36 | 566287 | phldb1a |  |  | 17082 | -0.036 | -0.2787 | No |
| 37 | 564545 | si:dkey-40c11.1 |  |  | 17126 | -0.037 | -0.2792 | No |
| 38 | 101882842 | si:dkey-45k15.1 |  |  | 17377 | -0.049 | -0.2859 | No |
| 39 | 406318 | clta |  |  | 17484 | -0.053 | -0.2880 | No |
| 40 | 406591 | exoc6 |  |  | 17492 | -0.053 | -0.2870 | No |
| 41 | 751760 | llgl1 |  |  | 17716 | -0.063 | -0.2926 | No |
| 42 | 100034367 | utrn |  |  | 17796 | -0.067 | -0.2935 | No |
| 43 | 565842 | pclob |  |  | 18142 | -0.083 | -0.3025 | No |
| 44 | 550129 | sept10 |  |  | 18262 | -0.088 | -0.3042 | No |
| 45 | 570923 | rims4 |  |  | 18921 | -0.119 | -0.3222 | No |
| 46 | 321590 | sept8a |  |  | 19001 | -0.121 | -0.3219 | No |
| 47 | 431748 | dbnla |  |  | 19153 | -0.129 | -0.3237 | No |
| 48 | 567832 | exoc3l1 |  |  | 19164 | -0.130 | -0.3210 | No |
| 49 | 554123 | sept3 |  |  | 19212 | -0.131 | -0.3195 | No |
| 50 | 553383 | spna2 |  |  | 19315 | -0.136 | -0.3196 | No |
| 51 | 100002110 | stxbp6l |  |  | 19526 | -0.145 | -0.3229 | No |
| 52 | 404604 | clasp2 |  |  | 19579 | -0.147 | -0.3211 | No |
| 53 | 563393 | rims2b |  |  | 19595 | -0.148 | -0.3182 | No |
| 54 | 100000597 | sept7b |  |  | 20105 | -0.171 | -0.3303 | No |
| 55 | 336381 | cotl1 |  |  | 20136 | -0.172 | -0.3273 | No |
| 56 | 100006671 | zgc:165653 |  |  | 20161 | -0.173 | -0.3241 | No |
| 57 | 393431 | zgc:63587 |  |  | 20871 | -0.208 | -0.3416 | No |
| 58 | 337243 | sept9a |  |  | 21278 | -0.229 | -0.3491 | No |
| 59 | 553451 | sptbn1 |  |  | 21336 | -0.233 | -0.3456 | No |
| 60 | 564600 | sept12 |  |  | 22004 | -0.267 | -0.3604 | No |
| 61 | 559353 | si:dkey-30c15.10 |  |  | 22400 | -0.290 | -0.3662 | No |
| 62 | 561905 | mlpha |  |  | 22511 | -0.297 | -0.3629 | No |
| 63 | 570275 | phldb1b |  |  | 23099 | -0.334 | -0.3736 | No |
| 64 | 563246 | exoc3l4 |  |  | 23228 | -0.343 | -0.3698 | No |
| 65 | 553301 | exoc3l2a |  |  | 23290 | -0.347 | -0.3638 | No |
| 66 | 322694 | sept6 |  |  | 23764 | -0.378 | -0.3700 | No |
| 67 | 563056 | hcls1 |  |  | 23829 | -0.382 | -0.3633 | No |
| 68 | 323320 | actn3b |  |  | 23907 | -0.386 | -0.3568 | No |
| 69 | 58040 | sptb |  |  | 23985 | -0.391 | -0.3503 | No |
| 70 | 563576 | si:dkey-245p14.4 |  |  | 24471 | -0.418 | -0.3559 | No |
| 71 | 100148002 | pcloa |  |  | 24935 | -0.443 | -0.3603 | No |
| 72 | 558037 | zgc:162239 |  |  | 25031 | -0.449 | -0.3530 | No |
| 73 | 83773 | dmd |  |  | 25168 | -0.457 | -0.3468 | No |
| 74 | 406723 | exoc7 |  |  | 25188 | -0.459 | -0.3369 | No |
| 75 | 561671 | si:ch211-106h4.9 |  |  | 25313 | -0.468 | -0.3301 | No |
| 76 | 567159 | tnfaip2a |  |  | 26029 | -0.532 | -0.3404 | No |
| 77 | 101883199 | si:dkey-196h17.9 |  |  | 26324 | -0.559 | -0.3368 | No |
| 78 | 798260 | si:dkey-40c11.2 |  |  | 26679 | -0.596 | -0.3343 | No |
| 79 | 767763 | exoc6b |  |  | 26763 | -0.605 | -0.3230 | No |
| 80 | 100003123 | si:dkey-27p18.5 |  |  | 28509 | -0.823 | -0.3590 | No |
| 81 | 560003 | erc1b |  |  | 30222 | -1.351 | -0.3819 | Yes |
| 82 | 565839 | exoc3l2b |  |  | 30237 | -1.358 | -0.3512 | Yes |
| 83 | 100004870 | exoc2 |  |  | 30650 | -1.633 | -0.3267 | Yes |
| 84 | 548606 | erc1a |  |  | 30852 | -1.813 | -0.2915 | Yes |
| 85 | 58037 | actn3a |  |  | 30981 | -1.946 | -0.2509 | Yes |
| 86 | 792259 | sept15 |  |  | 31355 | -2.543 | -0.2043 | Yes |
| 87 | 334015 | exoc3 |  |  | 31457 | -2.792 | -0.1435 | Yes |
| 88 | 557239 | antxr2a |  |  | 31582 | -3.209 | -0.0738 | Yes |
| 89 | 541494 | sept9b |  |  | 31689 | -3.641 | 0.0063 | Yes |
Table: GSEA details
[plain text format]

  

Fig 2: CELL\_CORTEX\_PART(GO:0044448)
  
Blue-Pink O' Gram in the Space of the Analyzed GeneSet

  

Fig 3: CELL\_CORTEX\_PART(GO:0044448): Random ES distribution
  
Gene set null distribution of ES for
 **CELL\_CORTEX\_PART(GO:0044448)**
